# Polymicrobial retroperitoneal abscess by *Salmonella enteritidis* and *Bacteroides fragilis* unmasking cecal cancer

**DOI:** 10.1590/0037-8682-0446-2025

**Published:** 2026-02-06

**Authors:** Chee Yik Chang

**Affiliations:** 1 Hospital Sultanah Aminah, Medical Department, Infectious Diseases Unit, Johor Bahru, Johor, Malaysia.

**FIGURE 1:**
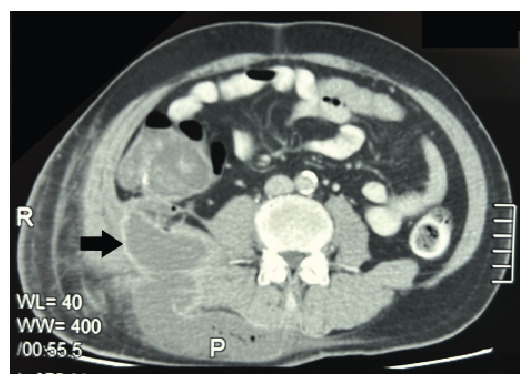
CT scan of the abdomen and pelvis showing a collection in the right retroperitoneal region involving the right quadratus lumborum communicating with a cecal tumor.

A 52-year-old man presented with a 5-day history of swelling on the right side of his back without fever, altered bowel habits, or rectal bleeding. Examination revealed a large, non-tender swelling measuring 15 × 10 cm on the right mid-to-lower back. Computed tomography of the thorax, abdomen, and pelvis showed a right retroperitoneal collection involving the quadratus lumborum muscle (7.7 × 18.9 × 14.3 cm) and a cecal mass (5.4 × 5.4 × 7.1 cm) with a colocutaneous fistula **(** Figure 1 **)**. The patient underwent incision and drainage of the abscess, followed by creation of a left ileostomy. Colonoscopy revealed a fungating cecal mass, which was confirmed as an adenocarcinoma on histopathological examination. *Salmonella enteritidis* and *Bacteroides fragilis* were isolated from the pus, both of which were susceptible to ampicillin-sulbactam. He received intravenous ampicillin-sulbactam (3 g every 6 h) for four weeks, which resulted in radiological resolution of the abscess.

Retroperitoneal abscesses and colocutaneous fistula are rare manifestations of colorectal cancer^1^ . Infection with *S. enteritidis* and *B. fragilis* has been linked to colorectal carcinogenesis through mechanisms such as intestinal dysbiosis, chronic inflammation, immune evasion, and activation of pro-tumorigenic signaling pathways^2^. Furthermore, *S. enteritidis* can cause life-threatening complications such as colonic perforations and pancytopenia^3^. Prompt surgical drainage and targeted antimicrobial therapy led to a complete recovery. The patient was subsequently scheduled for the definitive surgical removal of the cecal tumor.
